# (*Z*,*Z*)-*N*′′-[Amino­(pyrazin-2-yl)methyl­ene]pyrazine-2-carbohydrazonamide

**DOI:** 10.1107/S1600536809027834

**Published:** 2009-07-22

**Authors:** Ya-Wen Zhang, Jian-Quan Wang, Lin Cheng

**Affiliations:** a School of Chemistry and Chemical Engineering, Southeast University, Nanjing 211189, People’s Republic of China

## Abstract

The title compound, C_10_H_10_N_8_, resides on a crystallographic symmetry center and features an essentially planar mol­ecule [r.m.s. deviation  = 0.278 (1) Å]. In the C=N—N=C fragment, the C=N distance is 1.3017 (18) Å and the N—N distance is 1.403 (2) Å. In the crystal, adjacent mol­ecules are linked by N—H⋯N hydrogen bonds into a three-dimensional network.

## Related literature

For related structures, see: Armstrong *et al.* (1998 ▶), Xu *et al.* (2006 ▶), Shi *et al.* (2008 ▶).
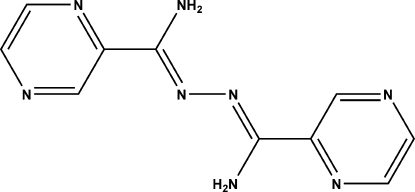


## Experimental

### 

#### Crystal data

C_10_H_10_N_8_
*M* *_r_* = 242.26
Monoclinic, 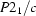

*a* = 8.6576 (16) Å
*b* = 6.6685 (12) Å
*c* = 9.6162 (18) Å
β = 97.682 (4)°
*V* = 550.19 (18) Å^3^
*Z* = 2
Mo *K*α radiation
μ = 0.10 mm^−1^
*T* = 295 K
0.30 × 0.19 × 0.16 mm

#### Data collection

Bruker SMART CCD diffractometer
Absorption correction: multi-scan (*SADABS*; Sheldrick, 2000 ▶) *T*_min_ = 0.970, *T*_max_ = 0.984
1946 measured reflections
946 independent reflections
844 reflections with *I* > 2σ(*I*)
*R*_int_ = 0.013

#### Refinement

*R*[*F* ^2^ > 2σ(*F* ^2^)] = 0.038
*wR*(*F* ^2^) = 0.108
*S* = 1.05
946 reflections
90 parameters
H atoms treated by a mixture of independent and constrained refinement
Δρ_max_ = 0.20 e Å^−3^
Δρ_min_ = −0.13 e Å^−3^

### 

Data collection: *SMART* (Bruker, 2000 ▶); cell refinement: *SAINT* (Bruker, 2000 ▶); data reduction: *SAINT*; program(s) used to solve structure: *SHELXS97* (Sheldrick, 2008 ▶); program(s) used to refine structure: *SHELXL97* (Sheldrick, 2008 ▶); molecular graphics: *SHELXTL* (Sheldrick, 2008 ▶); software used to prepare material for publication: *SHELXL97*.

## Supplementary Material

Crystal structure: contains datablocks I, global. DOI: 10.1107/S1600536809027834/bt5007sup1.cif

Structure factors: contains datablocks I. DOI: 10.1107/S1600536809027834/bt5007Isup2.hkl

Additional supplementary materials:  crystallographic information; 3D view; checkCIF report

## Figures and Tables

**Table 1:** Hydrogen-bond geometry (Å, °)

*D*—H⋯*A*	*D*—H	H⋯*A*	*D*⋯*A*	*D*—H⋯*A*
N3—H3*B*⋯N2^i^	0.906 (18)	2.280 (18)	3.0539 (18)	143.2 (14)
N3—H3*C*⋯N4^ii^	0.892 (19)	2.405 (18)	3.1587 (18)	142.3 (16)

Symmetry codes: (i) 
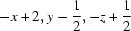
; (ii) 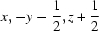.

**Fig. 1.**
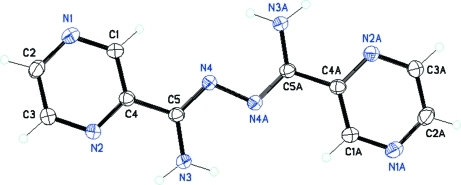
Structure of the title compound with 30% displacement ellipsoids. [Symmetry code: (A) 2-x, -1-y, -z.]

## supplementary crystallographic information

### Comment

The title compound can be regarded as a dihydrazidine with all atoms essentially coplanar and has now been shown to have *trans* geometry because of steric repulsion effect. In the C=N—N=C fragment, the C=N distance is 1.302 (2) Å, which is much shorter than the N—N distance of 1.403 (2) Å. All other C—N distances are 1.341 (2) Å, which are considered to have full double-bond character. Adjacent molecules are linked into a two-dimensional sheet by intermolecular N—H···N hydrogen bonds with the N···N distance of 3.054 (2) Å. Each molecule acts as double hydrogen-bond acceptors with the 2-positon N atoms of pyrazine rings and donors with the two amino groups.

### Experimental

A mixture of pyrazine-2-carbonitrile (0.210 g, 2 mmol), MnSO_4_.H_2_O (0.169 g, 1 mmol), hydrazine hydrate (80%, 2 ml) and anhydrous ethanol (6 ml) was heated in a 15 ml Teflon-lined autoclave at 393 K for 3 days, followed by slow cooling (5 K h^-1^) to room temperature. The resulting mixture was filtered and washed with 95% ethanol, and yellow block crystals were collected and dried in vacuum. Yield (0.32 g) 26.4%.

### Refinement

The H atom bonded to N were located in a difference map and   freely refined. Other H atoms were positioned geometrically and refined using a riding model with C—H = 0.93 Å and with *U*_iso_(H) = 1.2 *U*_eq_(C).

### Crystal data

**Table:**

C_10_H_10_N_8_	*F*(000) = 252
*M**_r_* = 242.26	*D*_x_ = 1.462 Mg m^−^^3^
Monoclinic, *P*2_1_/*c*	Mo *K*α radiation, λ = 0.71073 Å
Hall symbol: -P 2ybc	Cell parameters from 651 reflections
*a* = 8.6576 (16) Å	θ = 3.9–26.5°
*b* = 6.6685 (12) Å	µ = 0.10 mm^−^^1^
*c* = 9.6162 (18) Å	*T* = 295 K
β = 97.682 (4)°	Block, yellow
*V* = 550.19 (18) Å^3^	0.30 × 0.19 × 0.16 mm
*Z* = 2	

### Data collection

**Table:**

Bruker SMART CCD diffractometer	946 independent reflections
Radiation source: fine-focus sealed tube	844 reflections with *I* > 2σ(*I*)
graphite	*R*_int_ = 0.013
φ and ω scans	θ_max_ = 25.0°, θ_min_ = 2.4°
Absorption correction: multi-scan (*SADABS*; Sheldrick, 2000)	*h* = −10→4
*T*_min_ = 0.970, *T*_max_ = 0.984	*k* = −7→7
1946 measured reflections	*l* = −11→11

### Refinement

**Table:**

Refinement on *F*^2^	Primary atom site location: structure-invariant direct methods
Least-squares matrix: full	Secondary atom site location: difference Fourier map
*R*[*F*^2^ > 2σ(*F*^2^)] = 0.038	Hydrogen site location: inferred from neighbouring sites
*wR*(*F*^2^) = 0.108	H atoms treated by a mixture of independent and constrained refinement
*S* = 1.05	*w* = 1/[σ^2^(*F*_o_^2^) + (0.0659*P*)^2^ + 0.0834*P*] where *P* = (*F*_o_^2^ + 2*F*_c_^2^)/3
946 reflections	(Δ/σ)_max_ < 0.001
90 parameters	Δρ_max_ = 0.20 e Å^−^^3^
0 restraints	Δρ_min_ = −0.13 e Å^−^^3^

### Special details

**Table:**

Geometry. All e.s.d.'s (except the e.s.d. in the dihedral angle between two l.s. planes) are estimated using the full covariance matrix. The cell e.s.d.'s are taken into account individually in the estimation of e.s.d.'s in distances, angles and torsion angles; correlations between e.s.d.'s in cell parameters are only used when they are defined by crystal symmetry. An approximate (isotropic) treatment of cell e.s.d.'s is used for estimating e.s.d.'s involving l.s. planes.
Refinement. Refinement of *F*^2^ against ALL reflections. The weighted *R*-factor *wR* and goodness of fit *S* are based on *F*^2^, conventional *R*-factors *R* are based on *F*, with *F* set to zero for negative *F*^2^. The threshold expression of *F*^2^ > σ(*F*^2^) is used only for calculating *R*-factors(gt) *etc*. and is not relevant to the choice of reflections for refinement. *R*-factors based on *F*^2^ are statistically about twice as large as those based on *F*, and *R*- factors based on ALL data will be even larger.

### Fractional atomic coordinates and isotropic or equivalent isotropic displacement parameters (Å^2^)

**Table:**

	*x*	*y*	*z*	*U*_iso_*/*U*_eq_	
C1	0.71263 (17)	−0.1470 (2)	−0.12888 (15)	0.0400 (4)	
H1A	0.7177	−0.2641	−0.1806	0.048*	
C2	0.61260 (19)	0.1595 (2)	−0.10119 (17)	0.0459 (4)	
H2A	0.5502	0.2669	−0.1353	0.055*	
C3	0.69636 (18)	0.1731 (2)	0.03029 (16)	0.0421 (4)	
H3A	0.6851	0.2866	0.0842	0.050*	
C4	0.80524 (15)	−0.1312 (2)	0.00078 (14)	0.0326 (4)	
C5	0.92285 (16)	−0.2845 (2)	0.05271 (14)	0.0322 (4)	
N1	0.61789 (15)	−0.0024 (2)	−0.18134 (13)	0.0462 (4)	
N2	0.79325 (14)	0.02862 (18)	0.08288 (12)	0.0372 (4)	
N3	1.00358 (16)	−0.2540 (2)	0.17988 (13)	0.0431 (4)	
H3B	1.071 (2)	−0.351 (3)	0.2148 (18)	0.045 (4)*	
H3C	0.974 (2)	−0.154 (3)	0.2320 (19)	0.050 (5)*	
N4	0.94266 (14)	−0.43331 (17)	−0.03069 (11)	0.0356 (4)	

### Atomic displacement parameters (Å^2^)

**Table:**

	*U*^11^	*U*^22^	*U*^33^	*U*^12^	*U*^13^	*U*^23^
C1	0.0420 (8)	0.0408 (8)	0.0350 (8)	0.0018 (6)	−0.0029 (6)	−0.0042 (7)
C2	0.0441 (9)	0.0435 (9)	0.0475 (9)	0.0069 (7)	−0.0038 (7)	0.0066 (7)
C3	0.0468 (9)	0.0355 (8)	0.0426 (9)	0.0047 (6)	0.0013 (7)	−0.0023 (7)
C4	0.0353 (8)	0.0329 (8)	0.0295 (7)	−0.0030 (6)	0.0039 (6)	0.0006 (6)
C5	0.0363 (8)	0.0316 (7)	0.0280 (7)	−0.0025 (6)	0.0017 (6)	0.0015 (6)
N1	0.0459 (8)	0.0486 (8)	0.0405 (8)	0.0040 (6)	−0.0078 (6)	0.0010 (6)
N2	0.0429 (7)	0.0339 (7)	0.0335 (7)	0.0024 (5)	−0.0002 (5)	−0.0018 (5)
N3	0.0534 (9)	0.0409 (8)	0.0317 (7)	0.0145 (6)	−0.0068 (6)	−0.0050 (6)
N4	0.0425 (7)	0.0312 (7)	0.0313 (7)	0.0042 (5)	−0.0016 (5)	−0.0001 (5)

### Geometric parameters (Å, °)

**Table:**

C1—N1	1.3219 (19)	C4—N2	1.3386 (18)
C1—C4	1.393 (2)	C4—C5	1.4815 (19)
C1—H1A	0.9300	C5—N4	1.3017 (18)
C2—N1	1.331 (2)	C5—N3	1.3405 (18)
C2—C3	1.374 (2)	N3—H3B	0.906 (18)
C2—H2A	0.9300	N3—H3C	0.893 (19)
C3—N2	1.3318 (19)	N4—N4^i^	1.403 (2)
C3—H3A	0.9300		
			
N1—C1—C4	122.66 (14)	C1—C4—C5	122.46 (13)
N1—C1—H1A	118.7	N4—C5—N3	125.59 (13)
C4—C1—H1A	118.7	N4—C5—C4	117.36 (12)
N1—C2—C3	122.11 (14)	N3—C5—C4	117.01 (13)
N1—C2—H2A	118.9	C1—N1—C2	116.02 (13)
C3—C2—H2A	118.9	C3—N2—C4	116.52 (12)
N2—C3—C2	122.01 (14)	C5—N3—H3B	117.6 (10)
N2—C3—H3A	119.0	C5—N3—H3C	118.3 (11)
C2—C3—H3A	119.0	H3B—N3—H3C	123.0 (15)
N2—C4—C1	120.49 (13)	C5—N4—N4^i^	111.61 (13)
N2—C4—C5	117.03 (12)		

Symmetry codes: (i) −*x*+2, −*y*−1, −*z*.

### Hydrogen-bond geometry (Å, °)

**Table:**

*D*—H···*A*	*D*—H	H···*A*	*D*···*A*	*D*—H···*A*
N3—H3B···N2^ii^	0.906 (18)	2.280 (18)	3.0539 (18)	143.2 (14)
N3—H3C···N4^iii^	0.892 (19)	2.405 (18)	3.1587 (18)	142.3 (16)

Symmetry codes: (ii) −*x*+2, *y*−1/2, −*z*+1/2; (iii) *x*, −*y*−1/2, *z*+1/2.
